# Machine Learning for Medical Imaging

**DOI:** 10.1155/2019/9874591

**Published:** 2019-04-28

**Authors:** Geng-Shen Fu, Yuri Levin-Schwartz, Qiu-Hua Lin, Da Zhang

**Affiliations:** ^1^Amazon.com, Cambridge, USA; ^2^Icahn School of Medicine at Mount Sinai, New York City, USA; ^3^Dalian University of Technology, Dalian, China; ^4^Beth Israel Deaconess Medical Center and Harvard Medical School, Boston, USA

Machine learning contains a set of methods, which allow a machine to learn meaningful patterns from data directly with minimal human interaction. The strength of a machine-learning technique is, in part, dependent on human knowledge. Such knowledge can help a machine to learn more efficiently through techniques like appropriate feature selection, transfer learning, and multitask learning. Through this symbiosis, machine learning has been successfully applied in many applications and achieves state-of-the-art performance [1–4]. More recently, machine-learning techniques have been applied to the field of medical imaging [5, 6].

With fast improving computational power and the availability of enormous amounts of data, deep learning [7] has become the default machine-learning technique that is utilized since it can learn much more sophisticated patterns than conventional machine-learning techniques. Unlike conventional machine-learning techniques, deep learning methods greatly simplify the feature engineering process and some have even been applied to raw data directly. This is especially important for the field of medical imaging analysis since it can take years of training to obtain adequate domain expertise for appropriate feature determination. Hence, this allows more researchers to exploit new ideas easier and faster.

Among all deep learning methods, convolutional neural networks (CNNs) are of special interest. By exploiting local connectivity patterns efficiently with shared weights, CNN, such as those utilized in the ImageNet competition [8], has quickly become a state-of-the-art method for image processing. Naturally, there are many recent works trying to apply CNN on medical image analysis [9, 10]. With methods like the rectified linear unit [11] and deep residual learning [12] alleviating issues such as the vanishing gradient problem, deeper models can be trained more efficiently and hence pushing deep learning to another level. However, there are still many remaining challenges, e.g., inconsistencies in data formats and lack of reliable training data, which need to be addressed. An active research topic is how to optimize the transfer of human knowledge to a machine-learning model.

This special issue focuses on applying machine-learning techniques to medical imaging data and covers topics from traditional machine-learning techniques, e.g., principle component analysis and support vector machine, to more recent ones, such as CNN. Transfer learning, which is used to address the issue of lacking sufficient medical image data for training, is also discussed. With the successful application of these techniques, papers in this special issue show progress on many fronts, such as the diagnosis of Alzheimer's disease and liver tumor segmentation. We hope that the readers will find these topics interesting.
